# Characterisation of radioiodinated flavonoid derivatives for SPECT imaging of cerebral prion deposits

**DOI:** 10.1038/srep18440

**Published:** 2015-12-16

**Authors:** Takeshi Fuchigami, Yuki Yamashita, Masao Kawasaki, Ayaka Ogawa, Mamoru Haratake, Ryuichiro Atarashi, Kazunori Sano, Takehiro Nakagaki, Kaori Ubagai, Masahiro Ono, Sakura Yoshida, Noriyuki Nishida, Morio Nakayama

**Affiliations:** 1 Department of Hygienic Chemistry, Graduate School of Biomedical Sciences, Nagasaki University, 1-14 Bunkyo-machi, Nagasaki 852-8521, Japan; 2 Faculty of Pharmaceutical Sciences, Sojo University, 4-22-1 Ikeda, Kumamoto 860-0082, Japan; 3 Department of Molecular Microbiology and Immunology, Graduate School of Biomedical Sciences, Nagasaki University, 1-12-4 Sakamoto, Nagasaki 852-8523, Japan; 4 Graduate School of Pharmaceutical Sciences, Kyoto University, 46-29 Yoshida Shimoadachi-cho, Sakyo-ku, Kyoto 606-8501, Japan

## Abstract

Prion diseases are fatal neurodegenerative diseases characterised by deposition of amyloid plaques containing abnormal prion protein aggregates (PrP^Sc^). This study aimed to evaluate the potential of radioiodinated flavonoid derivatives for single photon emission computed tomography (SPECT) imaging of PrP^Sc^. *In vitro* binding assays using recombinant mouse PrP (rMoPrP) aggregates revealed that the 4-dimethylamino-substituted styrylchromone derivative (SC-NMe_2_) had higher *in vitro* binding affinity (*K*_d_ = 24.5 nM) and capacity (*B*_max_ = 36.3 pmol/nmol protein) than three other flavonoid derivatives (flavone, chalcone, and aurone). Fluorescent imaging using brain sections from mouse-adapted bovine spongiform encephalopathy (mBSE)-infected mice demonstrated that SC-NMe_2_ clearly labelled PrP^Sc^-positive prion deposits in the mice brain. Two methoxy SC derivatives, SC-OMe and SC-(OMe)_2_, also showed high binding affinity for rMoPrP aggregates with *K*_i_ values of 20.8 and 26.6 nM, respectively. *In vitro* fluorescence and autoradiography experiments demonstrated high accumulation of [^125^I]SC-OMe and [^125^I]SC-(OMe)_2_ in prion deposit-rich regions of the mBSE-infected mouse brain. SPECT/computed tomography (CT) imaging and *ex vivo* autoradiography demonstrated that [^123^I]SC-OMe showed consistent brain distribution with the presence of PrP^Sc^ deposits in the mBSE-infected mice brain. In conclusion, [^123^I]SC-OMe appears a promising SPECT radioligand for monitoring prion deposit levels in the living brain.

Prion diseases, also called transmissible spongiform encephalopathies, are fatal neurodegenerative diseases characterised by the conversion of normal cellular prion proteins (PrP^C^) to abnormal PrP aggregates (PrP^Sc^). The human prion diseases, including Creutzfeldt–Jakob disease (CJD), variant CJD (vCJD), Gerstmann–Sträussler–Scheinker (GSS) disease, kuru and fatal familial insomnia are histopathologically typified by neuronal loss, astrocytosis, appearance of spongiform, and the presence of PrP^Sc^ deposits in the brain^1^,^2^. Although there have been considerable efforts in the development of therapeutic agents for prion diseases, there are no clinically efficacious drugs for them^3^. Detection of PrP^Sc^ at an early stage is considered important for the effective treatment against prion diseases because PrP^Sc^ has been found in the brain prior to the appearance of extensive clinical symptoms^4^,^5^. At present, post-mortem immunohistochemical analysis of PrP^Sc^ is still needed for definitive confirmation of prion diseases^6^,^7^. Recently, Atarashi *et al*. developed an ultrasensitive detection method for PrP^Sc^ from CSF called real-time quaking-induced conversion (RT-QUIC)^8^,^9^. Because of the high sensitivity (>80%) and selectivity (100%), this technique is a promising *ante mortem* diagnosis method for prion diseases. However, further clinical studies of large numbers of patients may be needed to establish the RT-QUIC as a standard definitive diagnosis method. On the other hand, nuclear medicine imaging such as single photon emission computed tomography (SPECT) and positron emission tomography (PET) may allow the direct visualisation of prion deposits composed of PrP^Sc^ in the living brain of prion disease patients. Hence, specific *in vivo* imaging agents for PrP^Sc^ deposits may be useful for monitoring the progression of these diseases and evaluating the efficacy of therapeutic interventions. Prion disease and Alzheimer’s disease have common histological features of insoluble amyloid formation from amyloid beta (A*β*) and PrP^Sc^, respectively^10^. Our laboratory and other research groups have thoroughly investigated the development of A*β* imaging agents for SPECT and PET^11^. Several radioligands for A*β* have been applied for imaging of prion deposits. [^125^I]IMPY has shown differential *in vitro* and *in vivo* brain distribution between scrapie-infected mice and age-matched control mice, but high background binding was observed^12^,^13^. 2-[4-(Methylamino) phenyl] benzothiazole (BTA-1) and 6-(2-fluoroethoxy)-2-(4-methylaminostyryl) benzoxazole (BF-168) fluorescently labelled the PrP^Sc^ plaques in the brain of scrapie-infected mice *in vivo*^14^,^15^. Clinical PET studies in GSS patients with [^11^C]2-(2-[2-dimethylaminothiazol-5-yl]ethenyl)-6-(2-[fluoro]ethoxy)benzoxazole ([^11^C]BF-227) demonstrated significant retention in cortical and subcortical brain regions, which are known as PrP^Sc^-rich areas, although further investigations may be necessary^16^. Accordingly, scaffolds of A*β* imaging agents may be useful for diagnosing prion diseases. We have developed radiolabelled flavonoid-related compounds, such as flavones (FLs)^17^,^18^, chalcones (CLs)^19^,^20^, aurones (ARs)^21^,^22^, and styrylchromones (SCs)^23^,^24^, as potential SPECT or PET imaging agents for A*β* plaques (Fig. 1).

We considered that these flavonoid derivatives have potential as diagnostic agents for prion diseases. Herein, we aimed to explore the feasibility of the flavonoid derivatives as imaging probes for detecting PrP^Sc^ in the living brain via *in vitro* experiments using recombinant mouse PrP protein (rMoPrP) and brain slices from mouse-adapted bovine spongiform encephalopathy (mBSE)-infected mice as prion disease models, followed by SPECT/CT studies in the mBSE-infected mice. We discovered that SPECT/CT imaging with a methoxy SC derivative [^123^I]SC-OMe successfully visualised the PrP^Sc^–positive regions in the brain of the prion disease mouse model.

## Results

### *In vitro* studies of flavonoid derivatives

**Figure 2:**
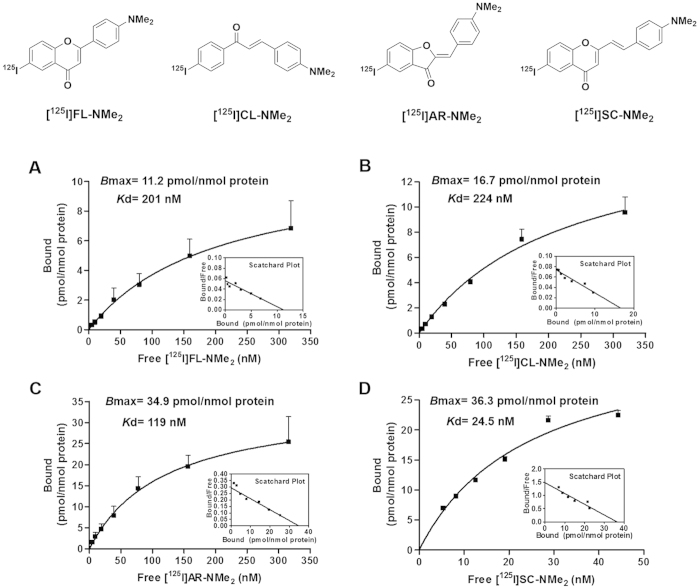
Saturation curves and Scatchard plots of the ^125^I-labelled flavonoid derivatives ([^125^I]FL-NMe_2_ (A), CL-NMe_2_ (B), AR-NMe_2_ (C), and SC-NMe_2_ (D)) binding to rMoPrP aggregates. *K*_d_ and *B*_max_ values were determined by saturation analysis using increasing concentrations of ^125^I-labelled flavonoids (6–350 nM). Values are the mean ± SEM of four to six independent measurements.

**Figure 3:**
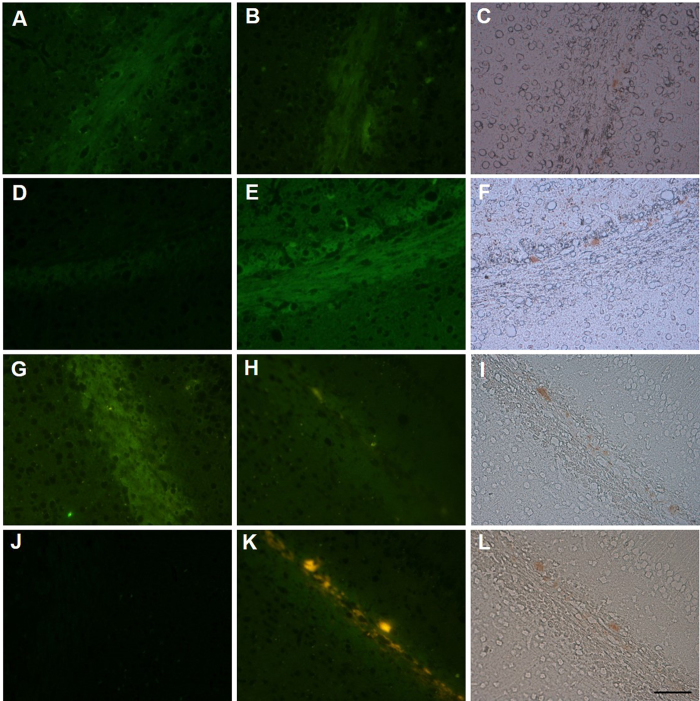
Fluorescence staining of flavonoid derivatives (FL-NMe_2_, CL-NMe_2_, AR-NMe_2_, and SC-NMe_2_) in brain sections from mock-infected mice (A,D,G,J) and brain sections from mBSE-infected mice (B,E,H,K). Labelled amyloid deposits of PrP^Sc^ were confirmed by immunohistochemical staining of each section using an anti-PrP antibody (**C**,**F**,**I**,**L**). Scale bar = 50 μm.

The rMoPrP aggregates were prepared as a PrP^Sc^ model according to previous reports^8^,^9^ for the *in vitro* binding assays of flavonoid derivatives to PrP^Sc^. Conversion of rMoPrP to *β*-sheet rich rMoPrP aggregates was confirmed by an increase in the fluorescence intensity of ThT (data not shown). We previously reported that flavonoid derivatives with a 4-dimethylamino group in a benzene ring showed the highest levels of binding affinity for A*β* aggregates among these series^17^,^19^,^21^,^23^. Accordingly, saturation binding assays of 4-dimethylamino-substituted flavonoid derivatives, including a flavone derivative [^125^I]FL-NMe_2_, a chalcone derivative [^125^I]CL-NMe_2_, an aurone derivative [^125^I]AR-NMe_2_ and a styrylchromone derivative [^125^I]SC-NMe_2_, for rMoPrP aggregates were evaluated to discover lead scaffolds of PrP^Sc^ imaging probes. As shown in Fig. 2, the binding of these ^125^I-labelled flavonoid derivatives to the rMoPrP aggregates demonstrated sigmoidal saturation curves and linear Scatchard plots that were fitted to single binding site models. [^125^I]FL-NMe_2_ (*K*_d_ = 201 nM, Fig. 2A) possessed moderate binding affinity for rMoPrP similar to that of [^125^I]CL-NMe_2_ (*K*_d_ = 246 nM, Fig. 2B), while [^125^I]AR-NMe_2_ showed higher affinity with a *K*_d_ value of 125 nM (Fig. 2C). [^125^I]SC-NMe_2_ showed a 3.5-fold higher binding affinity (*K*_d_ = 36.7 nM, Fig. 3D) compared with [^125^I]AR-NMe_2_. The rank order of their *B*_max_ values for rMoPrP aggregates was as follows: [^125^I]FL-NMe_2_ (11.2 pmol/nmol protein) < [^125^I]CL-NMe_2_ (16.7) < [^125^I]AR-NMe_2_ (34.9) < [^125^I]SC-NMe_2_ (36.3). These data indicate that [^125^I]SC-NMe_2_ has the highest binding affinity and capacity for rMoPrP aggregates among the four flavonoid derivatives. To evaluate the binding properties of flavonoid derivatives for PrP^Sc^ in brain tissue, the mBSE-infected mice were prepared as a mouse model of prion diseases^25^,^26^. Next, fluorescence staining of the four flavonoid derivatives was performed in brain slices from mBSE-infected mice. Brain slices from PBS-treated mice were used as a mock-infected group. Only background signals of the flavonoid derivatives (FL-NMe_2,_ CL-NMe_2,_ AR-NMe_2_ andSC-NMe_2_) were detected in brain sections from mock-infected mice (Fig. 3A,D,G,J, respectively). In contrast, SC-NMe_2_ clearly labelled PrP^Sc^ deposits in brain slices from mBSE-infected mice (Fig. 3K), while no significant fluorescence from the three other flavonoid derivatives was observed (Fig. 3B,E,H). Immunohistochemical analysis confirmed the presence of PrP^Sc^deposits in the adjacent sections of mBSE-infected mice (Fig. 3C,F,I,L). The PrP^Sc^-positive areas in mBSE-infected mice corresponded to the fluorescence signals obtained by SC-NMe_2_ (Fig. 3K,L).

### *In vitro* studies of SC derivatives

**Figure 4:**
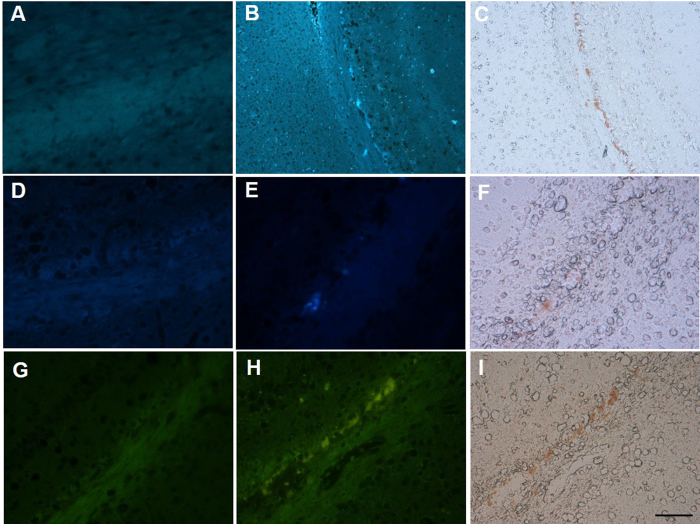
Fluorescence staining of SC derivatives (SC-OMe, SC-(OMe)_2_ and SC-NHMe) in the brain sections from mock-infected mice (A,D,G) and brain sections from mBSE-infected mice (B,E,H). Labelled amyloid deposits of PrP^Sc^ were confirmed by immunohistochemical staining of each section using an anti-PrP antibody (**C,F,I**). Scale bar = 50 μm.

**Figure 5:**
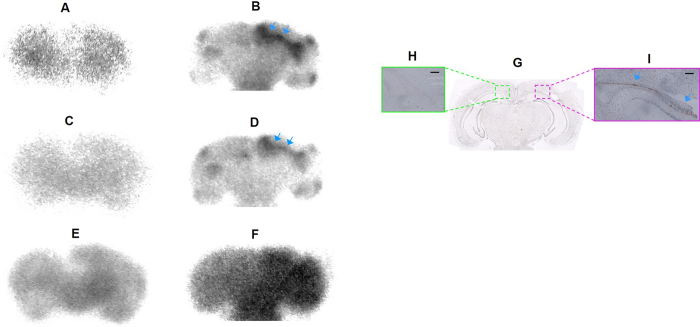
*In vitro* autoradiographic images of SC derivatives ([^125^I]SC-OMe, [^125^I]SC-(OMe)_2_ and [^125^I]SC-NHMe) in brain sections from mock-infected mice (A,C,E) and brain sections from mBSE-infected mice (B,D,F). Microscopic images of immunohistochemical staining for PrP^Sc^ in whole brain (**G**), the upper right hemisphere, which was the mBSE-inoculated region (**H**), and the contralateral site (**I**) of proximal sections from mBSE-infected mice. Arrows indicate PrP^Sc^-positive region. Scale bar = 100 μm.

Although SC-NMe_2_ showed high binding affinity for rMoPrP aggregates, as well as prion deposits, in the mBSE-infected mice, this radioligand has low brain uptake and slow washout from healthy mouse brain tissue *in vivo*^23^. We have developed amino- or alkoxy-substituted SC derivatives as A*β* imaging probes. Several of these exhibited high initial brain uptake with rapid clearance from the brain tissue of normal mice^23^,^24^. Therefore, we evaluated the feasibility of these SC derivatives as *in vivo* imaging probes for PrP^Sc^. We first examined the binding affinities of the SCs for rMoPrP aggregates using [^125^I]SC-NMe_2_ as a radioligand. The inhibition constants (*K*_i_) of the SCs for rMoPrP aggregates varied from 17.0 to 221 nM (Table 1). Methoxy derivative SC-OMe showed a *K*i value of 20.8 nM, while dimethoxy derivative SC-(OMe)_2_ had a slightly lower binding affinity (*K*_i_ = 26.6 nM). Replacing the 4-methoxy group of SC-OMe with a hydroxyl group (SC-OH, *K*_i_ = 35.0 nM) led to a slight decrease in binding affinity. The ethyleneoxy derivative SC-OEtOH showed an approximately six-fold lower binding affinity (*K*_i_ = 221 nM) than SC-OH. The primary amine derivative SC-NH_2_ exhibited a 2.8-fold lower binding affinity than SC-OMe, whereas the methylamino derivative SC-NHMe had comparable affinity (*K*_i_ = 17.0 nM) with SC-OMe. Because three SC derivatives, including SC-OMe, SC-(OMe)_2_ and SC-NHMe, showed high affinity for rMoPrP aggregates and had preferable lipophilicities (log P values; 2.15, 2.14 and 2.15, respectively^23^,^24^) for optimal passive brain entry *in vivo*^27^, we further evaluated neuropathological fluorescence staining of these SCs in brain slices of mBSE-infected and mock-infected mice. SC-OMe, SC-(OMe)_2_ and SC-NHMe showed no significant signals in mock-infected mouse brain slices (Fig. 4A,D,G). By contrast, clear fluorescence images of these compounds were detected in the brain sections of mBSE-infected mice (Fig. 4B,E,H), which corresponded to PrP^Sc^ deposit regions (Fig. 4C,F,I). Further *in vitro* autoradiography studies of ^125^I labelled SC derivatives ([^125^I]SC-OMe, [^125^I]SC-(OMe)_2_ and [^125^I]SC-NHMe) demonstrated the homogeneous distribution of radioactivity in the brain sections of mock-infected mice (Fig. 5A,C,E). [^125^I]SC-OMe and [^125^I]SC-(OMe)_2_ exhibited high signals in the right corpus callosum region of mBSE-infected mice (Fig. 5B,D), which spatially matched the distribution of PrP^Sc^ deposits (Fig. 5G,I). In contrast, these tracers showed no significant accumulation in the contralateral side of the brain (Fig. 5B,D), which showed no significant PrP^Sc^ deposits (Fig. 5G,H). Unfortunately, [^125^I]SC-NHMe displayed a high level of background and no significant accumulation of prion deposits in the mBSE-infected mouse brain (Fig. 5F).

### Evaluation of binding selectivity of SC-OMe to PrP^Sc^

SC-OMe showed potent binding affinity for rMoPrP aggregates and consistent distribution with PrP^Sc^-positive regions in mBSE-infected mice in the *in vitro* studies. Furthermore, [^125^I]SC-OMe has exhibited high initial brain uptake with favourable clearance from the brains of normal mice^24^. Accordingly, we further evaluated the usefulness of SC-OMe as an imaging probe for PrP^Sc^. We examined the binding selectivity of [^125^I]SC-OMe to PrP^Sc^ against PrP^C^. Dialysis methods have often been used to examine the binding properties of small molecular compounds and recombinant proteins^28^. Therefore, we evaluated the binding interactions between [^125^I]SC-OMe and native rMoPrP or rMoPrP aggregates with a dialysis method. The [^125^I]SC-OMe binding in 2.0 μM of rMoPrP aggregates was significantly higher (40.7%) than that in the same concentration of native rMoPrP (3.3%) (Supplemental Fig. 1). We further evaluated *in vitro* autoradiographs of [^125^I]SC-OMe in the brain sections of patients with AD, which demonstrated an inconsistent accumulation of [^125^I]SC-OMe with the existent Aβ plaques (Supplementary Fig. 2). These results indicated that [^125^I]SC-OMe binds to the PrP^Sc^ with high selectivity, as compared with PrP^C^ and A*β*.

### Small-animal SPECT/CT imaging of mBSE-infected mice

**Figure 6:**
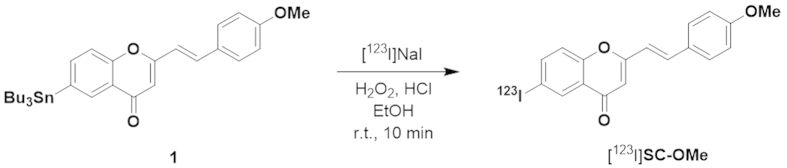
Radiosynthesis of [^123^I]SC-OMe.

**Figure 7:**
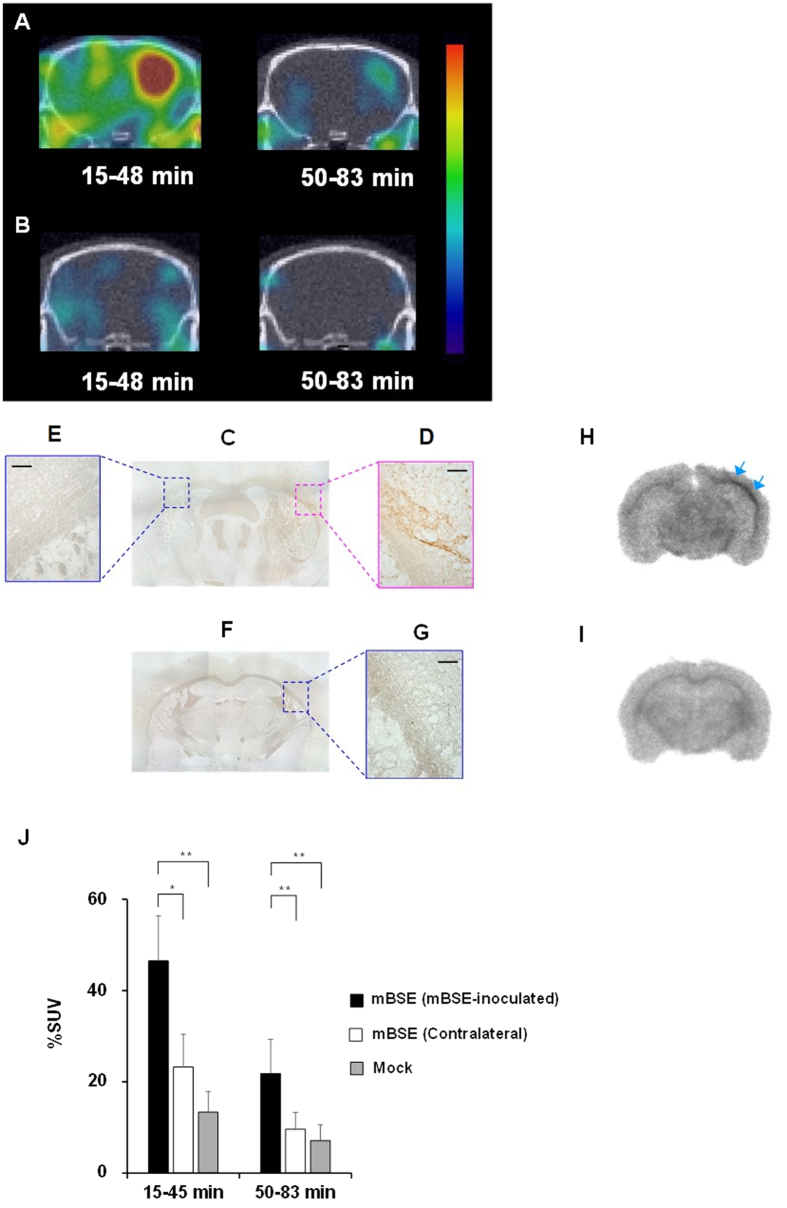
Representative composite SPECT/CT images of mBSE-infected (A) and mock-infected mice (B) over 15 to 48 min and 50 to 83 min after injection of [^123^I]SC-OMe. Microscopic images of immunohistochemical staining for PrP^Sc^ in whole brain (**C**), the upper right hemisphere, which was the mBSE-inoculated region (**D**), and the contralateral site (**E**) of brain tissue specimens from mBSE-infected mice. Immunohistochemical staining for PrP^Sc^ in whole brain (**F**) and the upper right hemisphere (**G**) of brain tissue specimens from mock-infected mice. Scale bar = 50 μm. *Ex vivo* autoradiography of corresponding brain slices from the same mBSE-infected (**H**) and mock-infected mice (**I**). Arrows indicate PrP^Sc^-positive region. The semiquantitative values obtained from the SPECT images are expressed as %SUV in the mBSE-inoculated upper right hemisphere, the contralateral site of the mBSE-infected mice and the PBS-injected ipsilateral hemisphere of mock-infected mice (**J**). Values are the means ± SD, n = 5. *P < 0.01, **P < 0.001 (ANOVA, Bonferroni’s test).

Metabolites of [^125^I]SC-OMe in plasma and brain tissues of normal mice at 30 min post-injection were analysed by radio-TLC. In plasma samples, a considerable amount of highly polar radiometabolites (83%) were observed and only 17% of the unchanged compound was detected. On the other hand, most of the parent compound remained unchanged (85%) in the brain homogenates (Supplementary Fig. 3), indicating that [^125^I]SC-OMe is stable in the brain and no significant metabolites entered the brain tissue. Therefore, we performed further preclinical small-animal SPECT studies with [^123^I]SC-OMe in mBSE-infected and mock-infected mice. For SPECT imaging, [^123^I]SC-OMe was synthesised by an iododestannylation reaction of corresponding tributyltin derivative **1** according to the method for the synthesis of [^125^I]SC-OMe, which yielded target [^123^I]SC-OMe at a radiochemical yield of 35–44% and a radiochemical purity of >98% (Fig. 6). Figure 7 shows representative SPECT/CT images in mice at an early period (15–48 min) and a late period (50–85 min) after intravenous injection of [^123^I]SC-OMe. The mBSE-infected mice showed significant [^123^I]SC-OMe accumulation in the mBSE-inoculated upper right hemisphere including cerebral cortex, hippocampus, and corpus callosum compared with the contralateral side at an early period. Although radioactivity in the brain decreased at a late period, a significant retention of radioactivity was observed in the upper right hemisphere (Fig. 7A). SPECT/CT images of mock-infected mice demonstrated moderate [^123^I]SC-OMe uptake in brain tissues at early periods, which decreased by late periods to only negligible signals (Fig. 7B). After SPECT/CT imaging, brain slices from mice were further characterised by immunohistochemical staining of PrP^Sc^. High [^123^I]SC-OMe signal areas in the upper right hemisphere of mBSE-inoculated regions were confirmed to be PrP^Sc^-positive areas (Fig. 7C,D), whereas PrP^Sc^ was absent in the contralateral brain hemisphere (Fig. 7C,E). There were no PrP^Sc^-positive areas in brain tissues from mock-infected mice (Fig. 7F,G). *Ex vivo* autoradiography of brain slices demonstrated significant [^123^I]SC-OMe accumulation in PrP^Sc^-positive regions and no significant accumulation was detected in the contralateral site of mBSE-infected mice (Fig. 7H). However, low signals of [^123^I]SC-OMe binding were observed in the mock-infected mouse brain (Fig. 7I). The semiquantitative %SUV values of SPECT images in the PrP^Sc^-positive upper right hemisphere of the mBSE-infected mouse were significantly higher (%SUV = 46.5) compared with those in the contralateral site (%SUV = 23.3, P<0.01) and the PBS-injected ipsilateral hemisphere of mock-infected mice (%SUV = 13.4, P < 0.001). At the late period, the [^123^I]SC-OMe binding in the right hemisphere of mBSE-infected mice (%SUV = 7.5) was still significantly higher than those in the contralateral site (%SUV = 3.7, P < 0.001) and the ipsilateral site of mock-infected mice (%SUV = 3.5, P < 0.001) (Fig. 7J). There was no significant difference in [^123^I]SC-OMe uptake between the contralateral hemisphere of mBSE-infected mice and brain tissue from mock-infected mice.

## Discussion

Nuclear medicine imaging of PrP^Sc^ in the living brain may be useful for monitoring the progression of these diseases and for the evaluation of the efficacy of therapeutic interventions at an early stage. There have been several reports on A*β* imaging agents being applied to prion imaging^12^,^13^,^14^,^15^,^16^. In particular, Okamura *et al*. reported on the consistent *in vitro* autoradiograms of [^18^F]BF-227 with PrP deposits in GSS brain sections. They also showed high [^11^C]BF-227 retention in PrP^Sc^-rich brain tissue from GSS patients using PET studies^16^; however, the tracer is known to be a nonspecific amyloid imaging agent^29^. We carried out detailed *in vitro* evaluations of flavonoid derivatives using rMoPrP aggregates as a PrP^Sc^ model^8^,^9^ and brain slices from mBSE-infected mice known as an animal model of prion diseases^25^,^26^. In addition, we evaluated their *in vivo* potential using SPECT/CT imaging and *ex vivo* autoradiography of mBSE-infected mice. To our knowledge, this study is the first to describe *in vivo* imaging of PrP^Sc^ in a rodent model of prion diseases using small-animal nuclear medicine imaging systems. We have demonstrated that SC derivatives can be applied to *in vivo* imaging probes for the detection of prion deposits in the brain. It should be noted that SPECT/CT studies with [^123^I]SC-OMe successfully visualised PrP^Sc^-positive regions in the mBSE-infected mouse brain.

*In vitro* binding studies suggested that SC derivatives may be the most promising candidate imaging probes for PrP^Sc^ among the four flavonoid derivatives (Figs 2, 3, 4, 5). We previously found that four 4-dimethylamino-substituted flavonoid derivatives (FL-NMe_2_, CL-NMe_2_, AR-NMe_2_, SC-NMe_2_) all showed high binding affinities for A*β* aggregates and clearly stained amyloid plaques in AD mouse model (*Tg 2576* mice) brains^17^,^19^,^21^,^23^. It is unclear why SC-NMe_2_ bound with the highest affinity to rMoPrP aggregates and prion deposits while other compounds had unsatisfactory binding properties for PrP^Sc^. Considering that styrylbenzoazole derivatives also showed binding affinity for prion deposits^15^, a styryl group directly binding to an aromatic ring may contribute to the interaction between SCs and the amyloid of the prion protein. PrP^Sc^ deposits were only detected close to the mBSE infection site in the mouse brain (Figs 3, 4, 5 and 7), which were fewer compared with A*β* deposits in the *Tg2576* mouse brain^19^. Therefore, it may be difficult to stain PrP^Sc^ deposits in the brain region of our mBSE-infected mouse model with some A*β* imaging agents. Because established radioligands for *in vivo* imaging of PrP^Sc^ have not yet been developed, there are no criteria for *K*_d_ and *B*_max_ values of compounds for rMoPrPaggregates in the screening process of prospective *in vivo* imaging probes for PrP^Sc^. Recently, Chen *et al*. reported that SPECT imaging with ^123^I-DRM106 successfully detected A*β* deposition in living aged transgenic mice. ^125^I-DRM106 exhibited a *K*_d_ value of 10.1 nM and a *B*_max_ value for A*β* (1–42) fibrils of 34.3 pmol/nmol^30^. Similarly, [^125^I]SC-NMe_2_ exhibited a *K*_d_ value of 24.5 nM and a *B*_max_ for rMoPrP aggregates of 36.3 pmol/nmol. Although the amyloid models differ, the results of our *in vitro* experiments of SC derivatives could provide one of the criteria for the development of *in vivo* imaging probes for PrP^Sc^. Among the SC derivatives, the methoxy derivatives (SC-OMe and SC-(OMe)_2_) and SC-NHMe showed relatively high affinity for rMoPrP aggregates, suggesting that electron-donating and lipophilic substituents in the 4-position of the 2-styryl group may be important for binding interaction with rMoPrP aggregates (Table 1). In particular, [^125^I]SC-OMe and [^125^I]SC-(OMe)_2_ labelled prion deposits in the brain sections from mBSE-inoculated mice by fluorescence microscopy (Fig. 4) and *in vitro* autoradiography (Fig. 5). Moreover, SPECT/CT imaging with [^123^I]SC-OMe and *ex vivo* autoradiography studies in mice revealed higher levels of tracer accumulation in PrP^Sc^-positive brain regions of mBSE-infected mice compared with PrP^Sc^-negative brain regions and the corresponding brain regions of mock-infected mice (Fig. 7). Importantly, our recent report and this study demonstrated that [^125^I]SC-OMe failed to detect A*β* plaques in *Tg2576* mouse brain sections^24^ and AD patient brain sections (Supplemental Fig. 2) by *in vitro* autoradiography, which suggested that [^123^I]SC-OMe could distinguish prion deposits from A*β* plaques. In addition, we confirmed that [^125^I]SC-OMe selectively bound to PrP^Sc^ rather than PrP^C^ (Supplemental Fig. 1). It should be taken into consideration that overall radioactivity levels in the brain of mBSE-infected mice were higher than those of mock-infected mice, indicating that the blood–brain barrier (BBB) of mBSE-infected mice was altered. In fact, several reports suggested that prion infection is related to BBB disruption^31^,^32^. Nevertheless, these results indicate that SPECT imaging using [^123^I]SC-OMe can be helpful in distinguishing PrP^Sc^-positive regions from PrP^Sc^-negative regions. Although further preclinical SPECT imaging studies of [^123^I]SC-OMe using various animal models of prion diseases are necessary, [^123^I]SC-OMe has exhibited higher selectivity for PrP^Sc^ than other previously reported amyloid imaging probes, and may be a prospective SPECT imaging probe for prion deposits. Such a probe can be used for further investigations into the mechanisms of prion diseases as well as development of therapeutic agents for these diseases both in basic investigations and clinical studies.

In conclusion, we found that a SC backbone can be used as a scaffold for *in vivo* imaging agents of PrP^Sc^. We discovered the radioiodinated SC-OMe exhibited high affinity for rMoPrP aggregates and high accumulation in PrP^Sc^ positive regions of the mBSE-infected mouse brain. Notably, [^123^I]SC-OMe allowed prion deposit regions in mBSE-infected mice to be visualised by small animal SPECT/CT imaging systems. Overall, we demonstrate that [^123^I]SC-OMe could be a potential SPECT imaging probe for visualisation of PrP^Sc^ in the living brain.

## Methods

### General

All reagents were commercial products and used without further purification unless otherwise indicated. [^125^I]NaI was obtained by MP Biomedicals (Costa Mesa, CA, USA). [^123^I]NaI was supplied by FUJIFILM RI Pharma Co., Ltd. (Tokyo, Japan). High-performance liquid chromatography (HPLC) analysis was performed on a Shimadzu HPLC system (LC-10AT pump with a SPD-10A UV detector, λ = 254 nm). An automated gamma counter with a NaI(Tl) detector (2470 WIZARD^2^, PerkinElmer, MA, USA) was used to measure radioactivity. 6-Iodo-4′-dimethyaminoflavone (FL-NMe_2_) and [^125^I]FL-NMe_2_ were prepared according to the literature^17^. (*E*)-3-(4-(Dimethylamino)phenyl)-1-(4-iodophenyl) prop-2-en-1-one (CL-NMe_2_) and [^125^I]CL-NMe_2_ were prepared as described previously^19^. 2-[(4-Dimethylaminophenyl)methylene]-5-iodo-3(2H)-benzofuranone (AR- NMe_2_) and [^125^I]AR-NMe_2_ were prepared in accordance with another study^21^. (*E*)-6- Tributylstannyl-2-(4-methoxystyryl)-chromone (**1**), (*E*)-6-Iodo-2-(4-methoxystyryl) chromone (SC-OMe), (*E*)-6-Iodo-2-(3,4-dimethoxystyryl) chromone {SC-(OMe)_2_}, (*E*)-6-Iodo-2-(4-hydroxylstyryl)-chromone (SC-OH), (*E*)-6-Iodo-2-(4- hydroxyethoxystyryl)-chromone (SC-OEtOH), [^125^I]SC-OMe and [^125^I]SC-(OMe)_2_ were prepared as described previously^24^. (*E*)-6-Iodo-2-(4-aminostyryl)-chromone (SC-NH_2_), (*E*)-6-Iodo-2-(4-(methylamino)styryl)-chromone (SC-NHMe), (*E*)-6-Iodo- 2-(4-(dimethylamino)styryl)-chromone (SC-NMe_2_), [^125^I]SC-NHMe and [^125^I]SC-NMe_2_ were prepared as described previously^23^.

### Radiosynthesis of [^123^I]SC-OMe

The [^123^I]SC-OMe was prepared using a similar procedure for [^125^I]SC-OMe^24^. In brief, 3% (v/v) H_2_O_2_ (100 μL) was added to a mixture of corresponding tributyltin derivative (1.0 mg/400 μL-EtOH), [^123^I]NaI (111–222 MBq, specific activity 11.1 GBq/nmol), and 1 M HCl (100 μL) in a sealed vial. The reaction was allowed to proceed at room temperature for 10 min and terminated by addition of saturated NaHSO_3_aq (0.5 mL). After alkalisation with 0.5 mL of saturated NaHCO_3_aq and extraction with ethyl acetate, the extract was evaporated to dryness. The crude products were purified by HPLC on a Cosmosil C_18_ column (Nacalai Tesque, 5C_18_-AR-II, 10 × 250 mm) with an isocratic solvent of CH_3_CN/H_2_O (7:3) at a flow rate of 4.0 mL/min.

### Preparation of rMoPrP aggregates

Expression of the rMoPrP and aggregation of rMoPrP were carried out as described previously^8^,^9^. In brief, a solution of rMoPrP (2.0 μM) in NaCl/HEPES buffer (50 mM HEPES/KOH, 300 mMNaCl, pH 7.5) was added to a 96-well plate to create a final volume of 200 μL. The plate was incubated at 37 °C for 72 h in a shaker-equipped plate reader (Infinite F200 fluorescence plate reader; Tecan, Männedorf, Switzerland) with repeated 30 s of shaking and 30 s of pause. To determine the conversion of rMoPrP to *β*-sheet rich rMoPrP aggregates, freshly prepared rMoPrP aggregates were co-incubated with 10 μM of thioflavin-T (ThT) at room temperature for 10 min. The increase in fluorescence intensity was measured using a plate reader at an excitation and emission wavelength of 440 and 485 nm, respectively.

### Binding assays using the rMoPrP aggregates

The saturation assays were performed by mixing an appropriate concentration of ^125^I-labelled flavonoid derivatives (0.15–8.75 kBq, 6–350 nM) and rMoPrP aggregates (100 nM) in NaCl/HEPES buffer (50 mM HEPES/KOH, 300 mM NaCl, pH 7.5) containing 20% (v/v) dimethyl sulfoxide (DMSO). After incubation for 2 h at room temperature, the mixture was then filtered through Whatman GF/B filters using a Brandel M-24 cell harvester. Each assay tube before filtration and the filters containing the bound ^125^I ligand were measured by an automatic gamma counter and the bound/free ratio of [^125^I]ligand was calculated. The dissociation constant (*K*_d_) and binding capacity (*B*_max_) of compounds were estimated by Scatchard analysis using PRISM4 (GraphPad Software Inc., CA, USA). For competitive binding assays, the mixture contained [^125^I]SC-NMe_2_ (0.02 nM), test compound (8.0 pM–12.5 μM), and rMoPrP aggregates (100 nM) in NaCl/HEPES buffer (pH 7.5) containing 20% (v/v) DMSO. After incubation for 2 h at room temperature, the mixture was filtered and the filters were measured using the gamma counter. Nonspecific binding was defined in the presence of 10 μM for nonradioactive SC-NMe_2_. Values for the half maximal inhibitory concentration (IC_50_) were determined from displacement curves of three independent experiments using PRISM4, and those for the inhibition constant (*K*_i_) were calculated using the Cheng–Prusoff equation.

### Animals

All animals were supplied by Kyudo Co., Ltd. (Saga, Japan). Experiments using animals were conducted in accordance with our institutional guidelines and were approved by the Nagasaki University Animal Care Committee.

### Preparation of mBSE-infected mice and brain tissue samples

The mBSE-infectious animal experiments were conducted under biosafety level 3 (BSL3) containment in accordance with institutional guidelines. mBSE-infected mice were prepared as reported previously^25^,^26^. In brief, the right brain hemispheres of male ddY mice (4W) were intracerebrally infected with 20 μL of mBSE. For mock-infected mice, 20 μL of phosphate-buffered saline (PBS) was inoculated into the right hemispheres of mice. Mice were monitored weekly until the appearance of clinical onset, which was defined as the presence of three or more of the following signs: greasy and/or yellowish hair, hunchback, weight loss, yellow pubes, ataxic gait and nonparallel hind limbs^26^. The animals with characteristic symptoms were used for SPECT studies or sacrificed for *in vitro* studies at 22–25 weeks post-infection. The animals for *in vitro* experiments were exsanguinated by transcardial perfusion with saline under ether anaesthesia, and their brains were subsequently removed. Sacrificed brain tissues were fixated in 10% (v/v) buffered formalin for 1 week, and then each sample was embedded in paraffin and cut into 3-μm-thick sections.

### Fluorescent imaging and immunohistochemical analysis of rMoPrP deposition

The sections from mBSE-infected and mock-infected mice were dewaxed and incubated with a 50% (v/v) EtOH solution containing the test compound (100 μM) for 1 h. The sections were washed in 50% (v/v) EtOH for 2 min, two times. The fluorescence images were collected by an Eclipse 80i microscope (Nikon Corp., Tokyo, Japan) using a V-2A filter set (excitation, 380–420 nm; dichromic mirror, 430 nm; longpass filter, 450 nm) or a B-2A filter set (excitation, 450–490 nm; dichromic mirror, 505 nm; longpass filter, 520 nm). After fluorescent imaging analysis, the tissues were washed with 50% (v/v) EtOH and autoclaved in 1.2 mM of HCl at 121 °C for 10 min and then the sections were treated with formic acid for 15 min. After blocking with 0.3% (v/v) H_2_O_2_ for 30 min, normal goat serum (1:20) was added for 30 min. The tissues were incubated overnight with SAF32 anti-PrP antibody (1:20). Following washing with Tris-HCl buffer including 0.05% (v/v) Tween 20, the slices were incubated with secondary anti-mouse biotinylated antibody for 1.5 h. The signal was visualised by a reaction with hydrogen peroxidase-activated diaminobenzidine.

### *In vitro* autoradiography in mouse brain sections

Each brain section was incubated in 40% (v/v) DMSO solution containing [^125^I]ligand (10 kBq, 0.02 nM) for 1 h. The slices were rinsed for 5 min, two times each, with 70% (v/v) DMSO solution, and subsequently dipped into cold water for 30 s. The sections were dried under a steam of cold air and placed in contact with imaging plates (BAS-MS 2040; Fujifilm Corp., Tokyo, Japan) for 24 h. Distribution of radioactivity on the plates were analysed using the Fluoro Image Analyzer (FLA5100; Fujifilm Corp.). Thereafter, serial sections were also analysed by immunohistochemical staining of PrP^Sc^ deposition as described above.

### Small-animal SPECT/CT imaging of mBSE-infected mice

SPECT/CT imaging studies of mBSE-infected mice (ddY, 23–25 weeks old, male, 38.4–45.9 g, n = 5) or mock-infected mice (ddY, 23–25 weeks old, male, 42.4–51.3 g, n = 5) were performed using Triumph combined PET/SPECT/CT systems (TriFoil Imaging Inc., CA, USA). Each mouse was administered [^123^I]SC-OMe (32.0–43.4 MBq) via tail vein injection. Immediately after injection, the mice were anaesthetised with 1.5% (v/v) isoflurane. SPECT image acquisitions were performed with a four-head γ-camera equipped with five pinhole collimators (diameter, 1.0 mm; focal length, 75 mm). SPECT data were acquired for 33 min (radius of rotation, 40 mm, rotation angle, 180°; projection number, 16; time per projection, 120 s) starting at 15 or 50 min after intravenous injection. The SPECT imaging was followed by CT image acquisition (X-ray source, 60 kV; 256 projections), with the animal in exactly the same position. The SPECT data were reconstructed using a 3D-ordered subset expectation maximisation (3D-OSEM) algorithm in FLEX SPECT software. The semiquantitative values obtained from the SPECT images are expressed as the percent standardised uptake values (%SUV), which was calculated as follows:




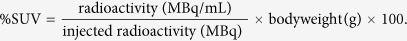




After SPECT/CT imaging, each mouse was sacrificed and the whole brain was frozen on dry ice/ethanol baths, followed by preparation of coronal sections (10 μm) using a cryostat microtome. Thereafter, the images of immunohistochemical staining of PrP^Sc^ and the autoradiograms of radioactivity in the brain sections were obtained using the same methods as described above.

### Statistical analysis

One-way analysis of variance followed by the post hoc tests using Bonferroni’s correction were used for analysis of significant differences for the %SUV values of SPECT images in the mouse brain tissues. A *P* value <0.05 was considered statistically significant.

## Additional Information

**How to cite this article**: Fuchigami, T. *et al*. Characterisation of radioiodinated flavonoid derivatives for SPECT imaging of cerebral prion deposits. *Sci. Rep*. **5**, 18440; doi: 10.1038/srep18440 (2015).

## Supplementary Material

Supplementary Information

## Figures and Tables

**Figure 1:**
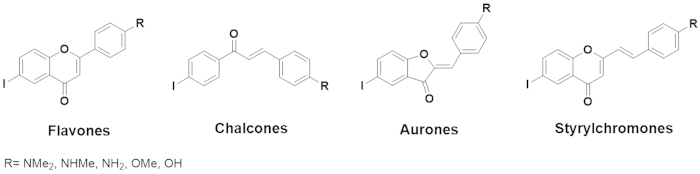
Chemical structures of flavonoid derivatives as A*β* imaging probes.

**Table 1:**
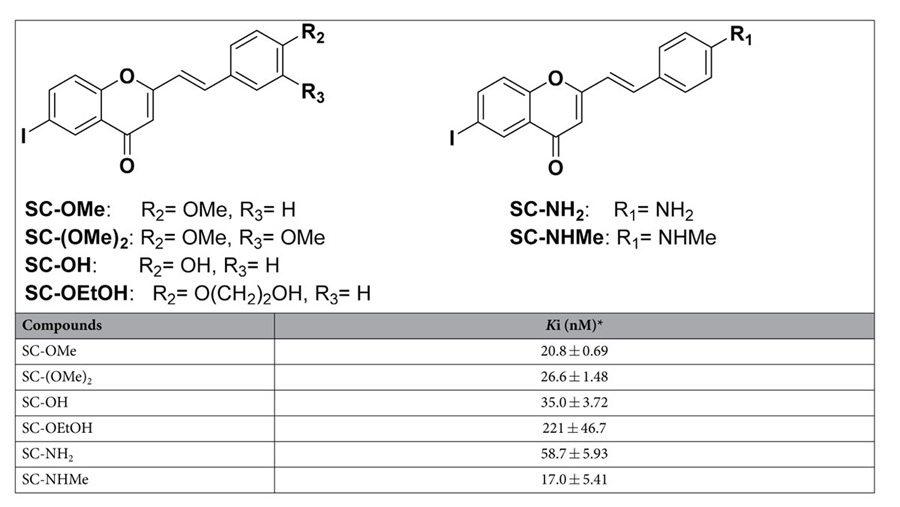
Inhibition constants (*K*_i_) of SC derivatives for rMoPrP aggregates.

^*^Ki values of SC derivatives were determined using [^125^I]SC-NMe_2_ as the ligand in rMoPrP aggregates. Each value (mean ± SEM) was determined by three to six independent experiments.
