# Supplementary material for: Characterisation of radioiodinated flavonoid derivatives for SPECT imaging of cerebral prion deposits
Source: Sci Rep. 2015 Dec 16;5:18440. doi: 10.1038/srep18440 (PMC4680881; doi:10.1038/srep18440)
Supplement: Supplementary Information [file srep18440-s1.pdf]

Supplementary information

Characterisation of radioiodinated flavonoid derivatives for SPECTimaging of cerebral prion deposits

Takeshi Fuchigami[1,*], Yuki Yamashita[1], Masao Kawasaki[1], Ayaka Ogawa[1], Mamoru Haratake[1,2], Ryuichiro Atarashi[3], Kazunori Sano[3], Takehiro Nakagaki[3],Kaori Ubagai[3], Masahiro Ono[4], Sakura Yoshida[1], Noriyuki Nishida[3], Morio Nakayama[1,*]

1. Department of Hygienic Chemistry, Graduate School of Biomedical Sciences, Nagasaki University, 1-14 Bunkyo-machi, Nagasaki 852-8521, Japan
2. Faculty of Pharmaceutical Sciences, Sojo University, 4-22-1 Ikeda, Kumamoto 860-0082, Japan
3. Department of Molecular Microbiology and Immunology, Graduate School of Biomedical Sciences, Nagasaki University, 1-12-4 Sakamoto, Nagasaki 852-8523, Japan
4. Graduate School of Pharmaceutical Sciences, Kyoto University, 46-29 Yoshida Shimoadachi-cho, Sakyo-ku, Kyoto 606-8501, Japan

*** Corresponding authors**

T. Fuchigami

E-mail: t-fuchi@nagasaki-u.ac.jp

M. Nakayama

E-mail: morio@nagasaki-u.ac.jp

Supplementary methods

Binding assays using rMoPrP and rMoPrP aggregates in a dialysis method. The mixtures of [^{125}I]SC-OMe (5.0 kBq) and native rMoPrP or rMoPrP aggregates (2 μM) in NaCl/HEPES buffer (50 mM HEPES/KOH, 300 mM NaCl, pH 7.5) containing 10% (v/v) DMSO were incubated for 2 h at room temperature. Unbound radiotracer was removed by dialysis against the assay buffer for 1 h using a microdialysis tool with a molecular cutoff of 14 kDa (Micro-dialyzer TOR-14K, Nippon Genetics Co. Ltd., Tokyo, Japan). The radioactivity of each sample before and after dialysis was measured using the gamma counter to quantify the bound [^{125}I]SC-OMe for rMoPrP or rMoPrP aggregates. Student's *t* test was used for analysis of significant differences.

***In vitro* autoradiography in human AD brain sections.** *In vitro* autoradiography of [^{125}I]SC-OMe in the postmortem brain slices was performed according to the method used with mBSE-infected mice. The postmortem brain tissues from an autopsy-confirmed case of AD (73-year-old male) were obtained from BioChain Institute, Inc. The presence and localization of plaques on the sections was confirmed by immunohistochemical staining using the monoclonal antibody BC05 (Wako) as already reported[1].

***In vivo* metabolism experiments.** [^{125}I]SC-OMe(185 kBq) was injected intravenously via the tail vein into ddY mice (male, 5 weeks old, 25–30 g, n = 3). After 30 min, the mice were euthanized, blood was collected, and whole brains were removed. Plasma was separated from blood cells by centrifugation at 10000 rpm for 5 min at 4 °C. The plasma samples were added to ice-cooled CH_3CN (1.0 ml) and then centrifuged to precipitate the plasma protein. Whole brains were quickly removed, homogenized with ice-cooled CH_3CN (1.0 ml), and centrifuged. Each supernatant in CH_3CN extract was analysed by radio-TLC using $CHCl_3$/MeOH = 20:1 as a mobile phase.

Supplementary Figure legends

Supplementary Figure 1

Binding assay for [^{125}I]SC-OMe with native rMoPrP or rMoPrP aggregates with a dialysis method. Values are means ± SEM, n = 3. * P < 0.0001 (Student's *t* test).

Supplementary Figure 2

In vitro autoradiographic images of [^{125}I]SC-OMe in the hippocampal section from an AD patient (73-year-old man) (A) and immunohistochemical staining of the section using a monoclonal anti-Aβ antibody (B).

Supplementary Figure 3

Representative *in vivo* metabolism study of mouse plasma (A) and brain homogenates (B) at 30 min after administration of [^{125}I]SC-OMe.

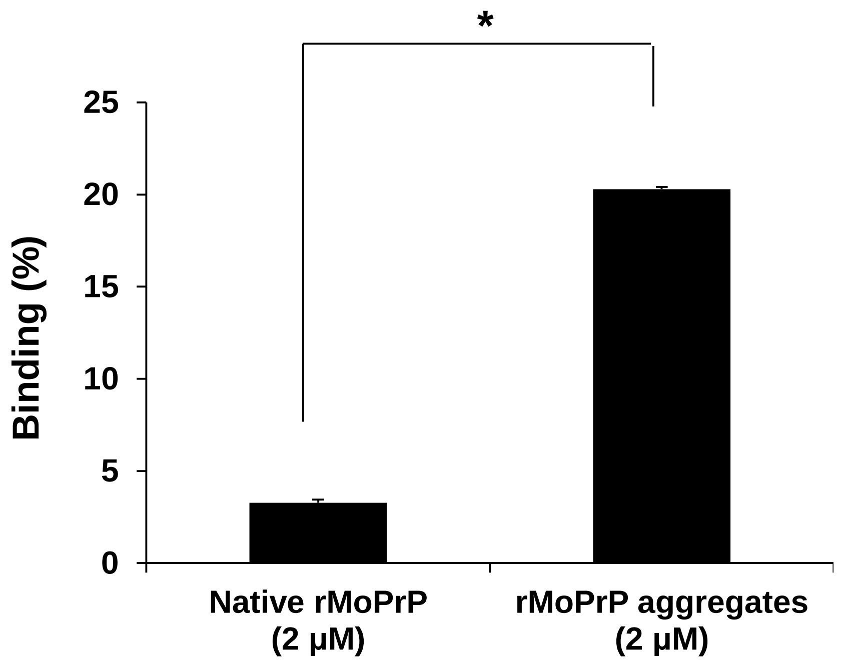

Supplementary Figure 1

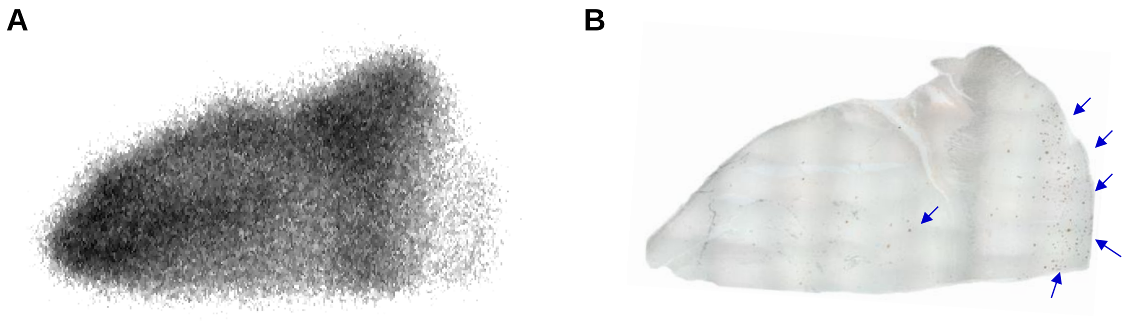

Supplementary Figure 2

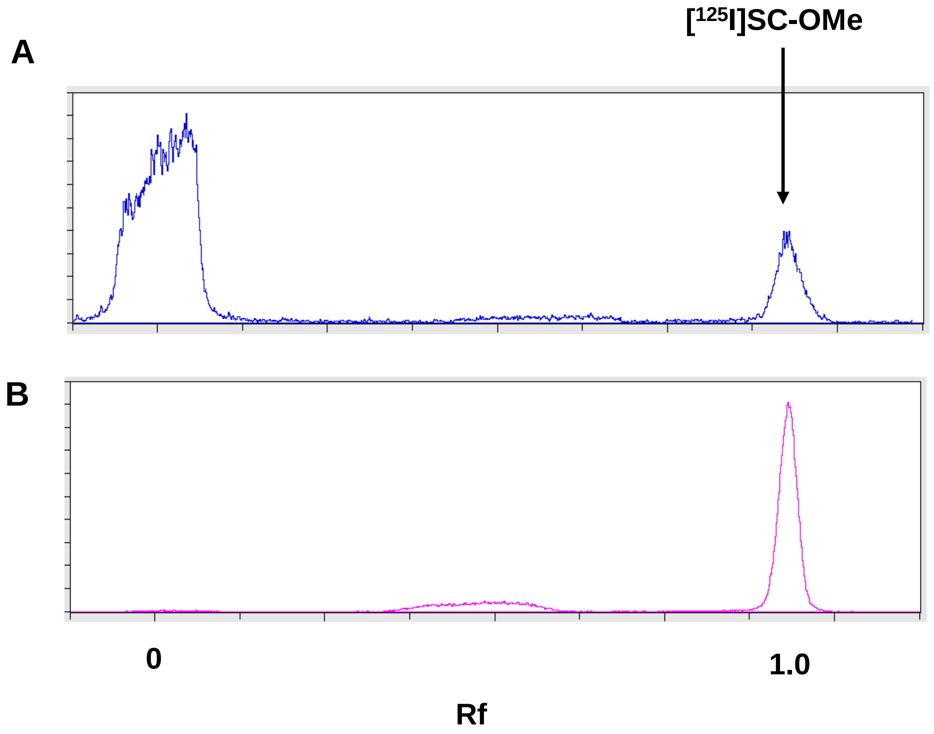

Supplementary Figure 3

References

1. Kung, M. P. et al. Binding of two potential imaging agents targeting amyloid plaques in postmortem brain tissues of patients with Alzheimer's disease. *Brain Res* **1025**, 98–105 (2004).
